# Whole-Exome Sequencing Characterized the Landscape of Somatic Mutations and Pathways in Colorectal Cancer Liver Metastasis

**DOI:** 10.1155/2019/2684075

**Published:** 2019-11-11

**Authors:** Liuxing Feng, Shifu Hong, Jin Gao, Jiayi Li

**Affiliations:** ^1^Department of Hepato-Biliary-Pancreato-Vascular Surgery, The First Affiliated Hospital of Xiamen University, Xiamen, Fujian, China; ^2^Department of Gastrointestinal Surgery, Cancer Hospital, The First Affiliated Hospital of Xiamen University, Teaching Hospital of Fujian Medical University, Xiamen, China; ^3^Department of Gastrointestinal Surgery, Xiamen Cancer Hospital, The First Affiliated Hospital of Xiamen University, Xiamen, Fujian 361003, China; ^4^Department of Medical Oncology, Cancer Hospital, The First Affiliated Hospital of Xiamen University, Teaching Hospital of Fujian Medical University, Xiamen, China

## Abstract

**Purpose:**

Liver metastasis remains the leading cause of cancer-related mortality in colorectal cancer. The mechanism of occurrence and development of liver metastasis from colorectal cancer is unclear.

**Methods:**

The primary tumor tissues and blood samples of 8 patients with liver metastasis of colorectal cancer were collected, followed by nucleic acid extraction and library construction. Whole-exome sequencing was performed to detect the genomic variations. Bioinformatics was used to comprehensively analyze the sequencing data of these samples, including the differences of tumor mutation burden, the characteristics of gene mutations, and signaling pathways.

**Results:**

The results showed that the top three genes with the highest mutation frequency were *TP53*, *APC*, and *KRAS*. Tumor mutation burden of this study, with a median of 8.34 mutations per MB, was significantly different with The Cancer Genome Atlas databases. Analysis of molecular function and signaling pathways showed that the mutated genes could be classified into five major categories and 39 signaling pathways, involving in Wnt, angiogenesis, P53, Alzheimer disease-presenilin pathway, notch, and cadherin signaling pathway.

**Conclusions:**

In conclusion, we identified the extensive landscape of altered genes and pathways in colorectal cancer liver metastasis, which will be useful to design clinical therapy for personalized medicine.

## 1. Introduction

Colorectal cancer (CRC) is the third most common type of malignancy and leading cause of cancer-related death worldwide [1]. Metastasis is still the main cause of cancer-related morbidity, and mortality of colorectal cancer due to liver metastasis accounts for about 25% [2]. Although, early detection and prevention or surgical resection of primary and metastatic lesions can reduce the risk of CRC and improve survival of CRC [3–5], metastatic CRC is still the leading cause of cancer-related deaths, and treatment options are not as selective.

A previous study suggested that the frequency rates of mutations such as *KRAS*, *NRAS*, *BRAF*, and *PIK3CA* in CRC differ among population [6]. *AMER1* is a frequently mutated gene in CRC comprising 553 samples [7]. *TMEM9*, as a novel human transmembrane protein, transactivated by *β*-catenin functions as a positive feedback regulator of WNT signaling in CRC and mTOR signaling, has been suggested to be an important factor involved in tumorigenesis [8, 9]. Therefore, a better understanding of the biological and phenotypic evolution of CRC and its molecular and genetic mechanisms during the transfer process is crucial.

To further investigate the genetic characteristics of colorectal cancer liver metastasis (CRLM), we performed whole-exome sequencing (WES) in 8 patients with CRLM. Somatic mutations, tumor mutation burden (TMB), molecular functions of mutational genes, and signaling pathways were analyzed. It is expected to provide clinical help for the treatment of patients with liver metastasis from colorectal cancer.

## 2. Patients and Methods

### 2.1. Patient Specimen Acquisition

Blood and primary CRC tumor tissue samples were collected from 8 patients with CRLM in the Oncology Department of the First Affiliated Hospital of Xiamen University during the period May 2018 to November 2018. Informed written consent was obtained from all patients before inclusion in the study. Respective tumor tissue samples which were a histologically confirmed adenocarcinoma by two molecular pathologists matched with the inclusion criteria. The study was conducted in accordance with the Helsinki Declaration and was approved by the Ethics Committee of the First Affiliated Hospital of Xiamen University [10].

### 2.2. DNA Extraction

DNA was extracted from serial sections cut from tumor samples and matched peripheral blood leukocytes as germline DNA control. The cases with tumor cell populations were estimated by pathologists to ensure more than 70% of cells were tumor cells. The DNA was isolated from the FFPE and blood samples using the DNeasy Blood and Tissue Kit (69504, QIAGEN, Venlo, Netherlands) according to the manufacturer's instructions. DNA quantity was assessed by using Agilent's Bioanalyzer (USA).

### 2.3. Whole-Exome Sequencing and Data Processing

The targeted capture pulldown and exon-wide libraries from genomic DNA were generated through the xGen® Exome Research Panel (Integrated DNA Technologies, Inc., Illinois, USA) and the TruePrep DNA Library Prep Kit V2 for Illumina (#TD501, Vazyme, Nanjing, China). The sequences of captured libraries were performed as pair-end reads on sequences on the Illumina HiSeq 2500 platform. Sequencing reads were processed and mapped to the reference GRCh37/hg19 human genome assembly and to the identified germline variations. Further local rearrangements were performed to improve the alignment of individual reads [11].

### 2.4. Variant Annotation and Mutation Signature Analysis

Somatic mutations identification and indels were annotated through Mutect [12] and Somatic Indel Detector [13]. The variant data were annotated using ANNOVAR [14] and Oncotator [15] and converted to MAF files by maf tools [16]. The cancer driver genes were analyzed using Intogen [17], including Oncodrive FM and Oncodrive CLUST. Both tools detect signals of positive selection, which appear in genes whose mutations are selected during tumor development and are therefore likely drivers. The landscape of top driver mutation spectrum predicted by Intogen for tumors was visulized via R Script, including mutation rate and mutation subclass/subtypes (filtering cutoff, ONCODRIVE FM *P* value ≤ 0.1).

### 2.5. Statistical Analyses

All the correlate clinical and biological variables were employed using the SPSS Statistics 22.0 package and ggpubr package [18] in R [19] by means of Fisher's test or a nonparametric test when necessary. The Kruskal–Wallis test was used to analyze whether TMB differ between different data sets.

## 3. Results

### 3.1. Patient Characteristics

We collected tumor tissue and matched blood from 8 patients with CRLM at the time of diagnosis, including 5 males and 3 females, with an average age of 66.6 years (range, 46–83 years). One of the patients was a former smoker, and the other seven were nonsmokers. Additionally, one male patient was also alcoholic. According to the anatomical classification system, 75.0% (6/8) of samples were classified as left hemicolon carcinoma, and the other 2 patients were right hemicolon carcinoma. All the patients were in stage IV and treated with chemotherapy. 37.5% (3/8) of the patients had a history of chronic disease, including diabetes, hypertension, coronary heart disease, and hyperuricemia. No patients received radiation therapy before surgery. The detailed clinical characteristics of the patients are shown in Table 1 and Supplemental [Supplementary-material supplementary-material-1].

### 3.2. Whole-Exome Sequencing and Identification of Somatic Mutations

**Figure 1:**
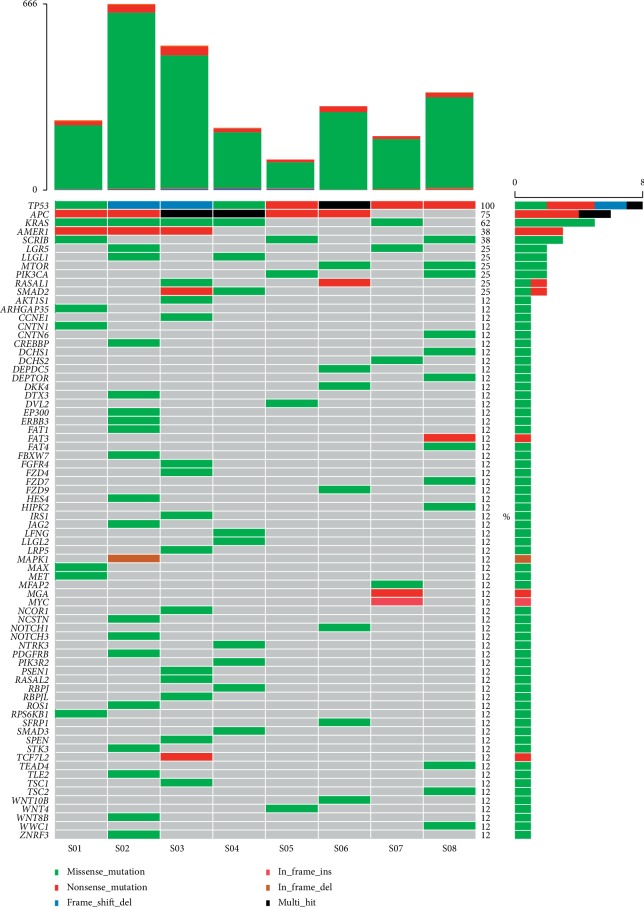
Landscape of somatic mutations in CRLM. The different colored tables represent different types of mutations (middle bars). We also calculated somatic mutations SNV using only somatic nonsynonymous mutations sequenced with WES for each sample (top bars), and the right bars represent the absolute number of mutations observed per gene across all samples.

We performed WES on DNA from 8 tumor tissues along with blood matched and then analyzed successfully with a mean depth of 244x. Somatic mutations were identified by comparing significant changes in nonreference alleles in the tumor and control groups. Overall, 1151 nonsynonymous single nucleotide variants (SNV) were identified (Supplemental [Supplementary-material supplementary-material-1]). An overview of the whole-exome sequencing results and the algorithm-generated arm-length copy number alterations are shown in Figure 1. Each gene with a nonsynonymous SNV was reviewed against known mutations identified in prior studies and subjected to Mutsig analysis. As shown in Figure 1, S02 have the most SNVs, following S03. We listed the top 75 genes based on the frequency of mutations. Among them, *TP53* (100%), *APC* (75%), and *KRAS* (62%) were the genes with the highest mutation rates. Missense mutation was the most common type of mutation, along with frame shift del, in frame ins, frame del, and so on (Figure 1).

**Figure 2:**
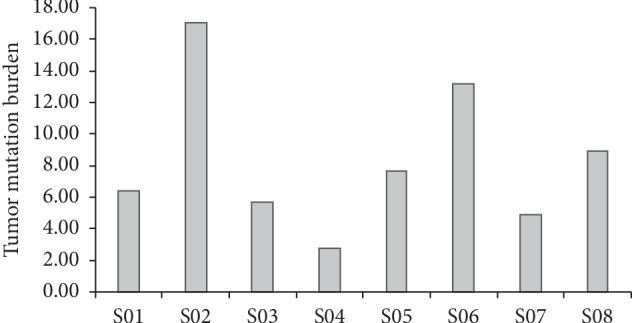
TMB analysis in CRLM patients.

We also calculated TMB using only somatic nonsynonymous mutations sequenced with WES. On the whole, we found that the TMBs of different samples were significantly different, with a median of 8.34 mutations per MB (range, 2.79–17.04 mutations/MB) (Figure 2).

**Figure 3:**
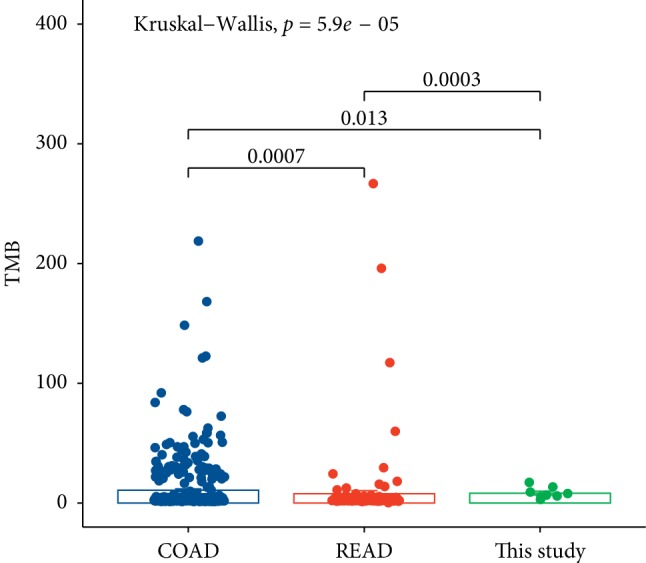
Comparison of TMB between the date in this study and COAD with READ by thwe Kruskal–Wallis test. The primary sites of COAD and READ are colon and rectum.

In order to compare the differences in TMB between CRLM and TCGA database (COAD and READ), we used the Kruskal–Wallis nonparametric test to test the anova of multiple groups of data after homogeneity of variance test (therefore, anova cannot be used) and found significant difference between multiple database cohorts (*P* = 5.9*e* − 05) (Figure 3).

### 3.3. The Landscape of Mutational Signatures

In principle, all types of mutations (such as substitutions, indels, and rearrangements) and any accessory mutation characteristic, for example, the sequence context of the mutation or the transcriptional chain where the mutation occurred, can be incorporated into the set of features by which a mutational signature is defined.

**Figure 4:**
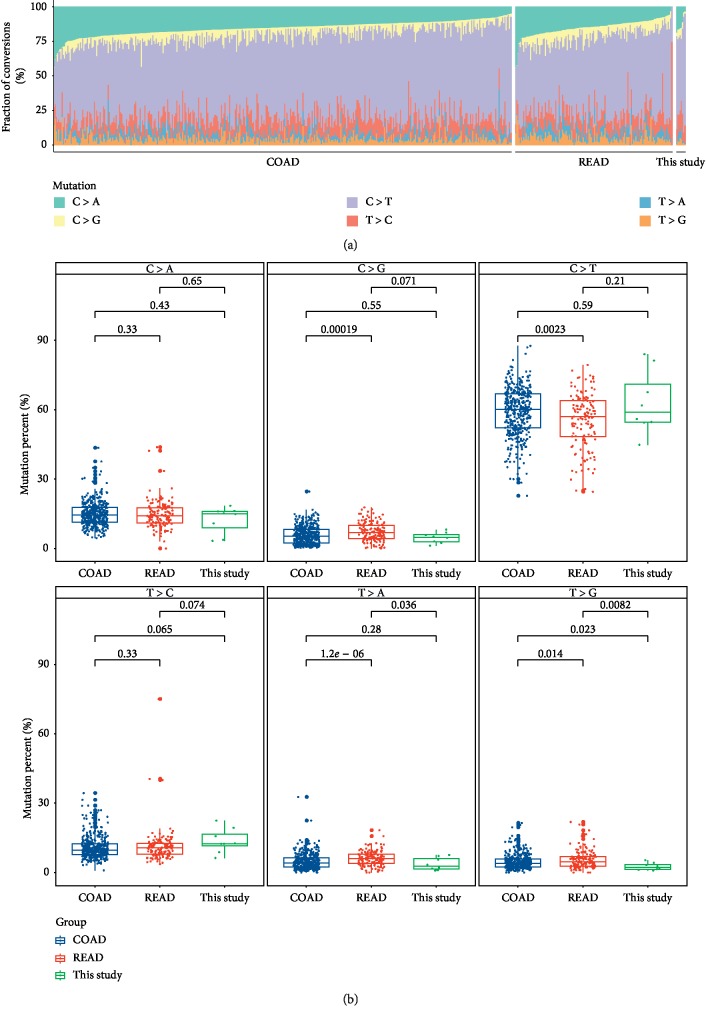
Mutational signature difference in multiple groups. (a) Transition and transversion proportions for six nucleotide changes. The stacked proportion bar chart is sorted by increasing the transition/transversion fraction. (b) Transition/transversion (Ti/Tv) ratios.

We extracted mutational signatures using base substitutions, and six classes of substitutions (C > A, C > G, C > T, T > C, T > A, and T > G) were referred to by the pyrimidine of the mutated Watson–Crick base pair. In this study, the six mutation types were compared with the TCGA database, and it was found that the proportion of these six mutation replacement types was roughly the same. The mutation percent of C > T was the highest in all substitutions, and this study has no significant difference with COAD and READ in this substitution. T > G substitution have significant difference between CRLM with COAD and READ (Figures 4(a) and 4(b)).

### 3.4. CRLM-Related Gene Molecular Function and Pathway Analyses

**Figure 5:**
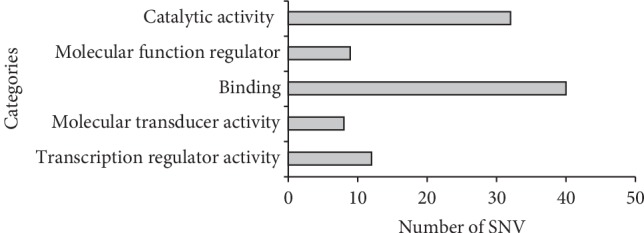
Go-slim molecular function by PANTHER for single nucleotide variants.

In order to further characterize the functions of mutational genes and their involved regulatory pathways, we used PANTHER classification system [20], an Ontology-Based Pathway Database Coupled with Data Analysis Tools. The results showed that molecular functions were divided into five categories, namely, binding, catalytic activity, molecular function regulator, molecular transducer activity, and transcription regulator activity. Of these, the category of binding (40) and catalytic activity (32) have the most function hits (Figure 5).

**Figure 6:**
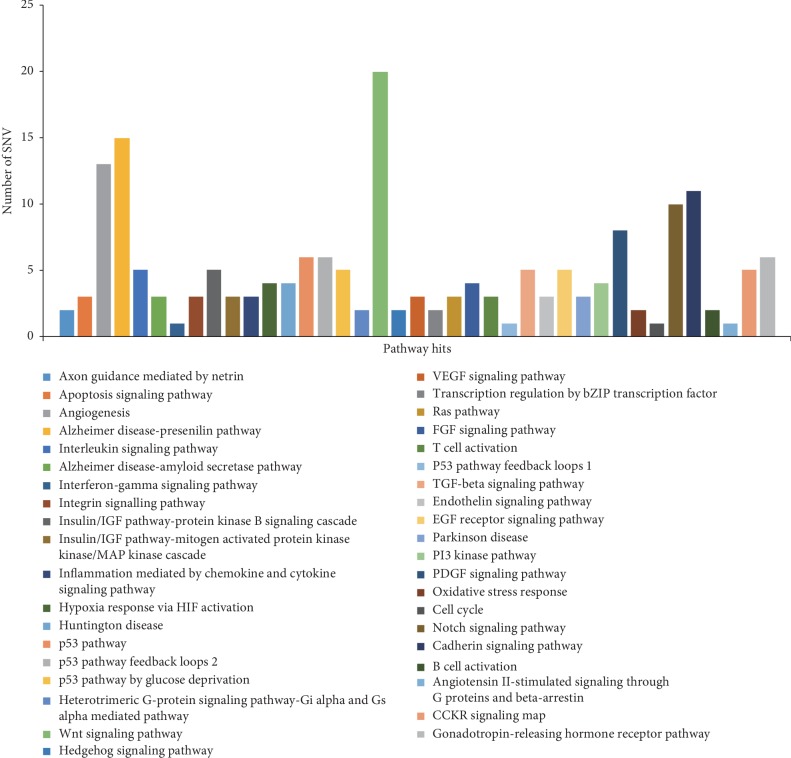
Analysis of pathways involving nonsynonymous genes in CRLM.

Through the PANTHER classification system pathway analysis, it was found that 74 pathway-related genes were involved in a total of 39 primarily signaling pathways, among which the pathways with higher frequency were Wnt signaling pathway (P00057), angiogenesis (P00005), P53 pathway (P00059), Alzheimer disease-presenilin pathway (P00004), notch signaling pathway (P00045), and cadherin signaling pathway (P00012). The other involved pathways and the genes involved in each pathway were referred to Figure 6 and Supplemental [Supplementary-material supplementary-material-1].

## 4. Discussion

CRC is the third most common malignancy in many countries and the second leading cause of cancer death. It develops from benign adenomatous polyp to invasive cancer, and nearly 50% of CRC patients develop into CRLM [21]. Without treatment, the median survival period of patients with colorectal liver metastasis is only 5–10 months, and the survival rate of over 5 years is less than 0.5% [22].

The molecular pathogenesis of CRC is related to a variety of genetic changes that result in abnormal activation of proto-oncogenes and inactivation of tumor suppressor genes [23]. We briefly described the characteristics of the CRLM by WES and had important insights into the genes and mechanisms of cancer occurrence and development. We found 1151 SNVs and the prevailing mutations were *APC*, *KRAS*, and *TP53* (Figure 1, Supplemental [Supplementary-material supplementary-material-1]), which is in accordance with data reported by The Cancer Genome Atlas Network [24]. Currently, there are dozens of biomarkers related to checkpoint inhibitors, among which TMB, PD-L1, and MSI/dMMR have been verified by phase III clinical trials and are widely used in clinical practice. Tumor mutation load (TMB) is a new biomarker for predicting PD-1/PD-L1 immune response [25]. Even though it has been reported that TMB ≥20 mutation/Mb (TMB-H) alone is not suitable for predicting the immunotherapy effect of each solid tumor type [26], we found that there was a significant difference in TMB between CRLM and colon and rectum, but the TMB did not exceed 20 mutations per MB (mean 8.34) (Figures 2 and 3). For different cancer types, the setting of high TMB threshold may need more clinical studies and a large number of patient information statistics.

The signatures can be understood as different mutation processes often generate different combinations of mutation types. Thousands of somatic mutations can be identified in a single cancer sample, making it possible to decipher the mutant signature, even if several mutations are operative [27]. The C > A mutational signature, is associated with smoking and chewing tobacco. Six classes of substitutions were extracted, and there was no significant difference in mutation percent between CRLM and colon with rectum cohorts (Figure 4). The genetic characteristics of liver metastasis may be more similar to that of the primary tumor, and the treatment strategy should be more similar to that of the primary tumor colorectal cancer.

Through pathway analysis, we found that oncogenes represented by *KRAS*, *PIK3CA*, *AKT1*, *PIK3R*, and tumor suppressor genes represented by *TP53*, *APC*, *EP300*, *CREBBP*, and *PIK3R1* were mutated, which may lead to changes in angiogenesis, TGF-*β*, Wnt signaling pathway, notch signaling pathway, and other pathways (Figure 6, Supplemental [Supplementary-material supplementary-material-1]). The pathway is complex, mainly reflected in the fact that one mutation gene is involved in multiple pathways [28], and various pathways are also cross-regulated, such as angiogenesis and notch signaling pathway.

## 5. Conclusion

Our study identified changes in driver gene mutations, TMB, and base Ti/Tv ratios in CRC with liver metastasis compared with rectal or colon cancer, although our study has some limitations, such as small sample size and lack of matched CRC liver metastasis samples. In conclusion, the current findings help define the genomic landscape of CRLM and identify specific pathways that are frequently altered, providing direction for research of targeted therapies against these tumors.

## Figures and Tables

**Table 1:** Patient characteristics.

Characteristic	No. of cases	Proportion (%)
Total number	*n* = 8	
Age, years (mean)	66.6 (46–83)	
Sex		
Male	5	62.5
Female	3	37.5
Smoking history		
Smoker	1	12.5
Nonsmoker	7	87.5
Drinking	1	12.5
Metastsis	7	87.5
Anatomical classification		
Right hemicolon	2	25.0
Left hemicolon	6	75.0
Stage		
IV	8	100
Chemotherapy	8	100

## Data Availability

All the related software and scripts used to support the findings of this study are available from the corresponding author upon request.

## Supplementary Materials

Supplementary Materials Supplemental Table 1: clinical characteristics of the patients. Supplemental Table 2: the number of SNV in the CRLM samples. Supplemental Table 3: list of pathway genes in CRLM.
