# Supplementary material for: Whole-Exome Sequencing Characterized the Landscape of Somatic Mutations and Pathways in Colorectal Cancer Liver Metastasis
Source: J Oncol. 2019 Nov 11;2019:2684075. doi: 10.1155/2019/2684075 (PMC6877969; doi:10.1155/2019/2684075)
Supplement: Supplementary Materials — Supplemental Table 1: clinical characteristics of the patients. Supplemental Table 2: the number of SNV in the CRLM samples. Supplemental Table 3: list of pathway genes in CRLM. [file 2684075.f1.zip › 2684075.f1/Supplemental Table 1.docx]

**Supplementary table 1 Patient clinical information**

| **Case** | **S01** | **S02** | **S03** | **S04** | **S05** | **S06** | **S07** | **S08** |
| --- | --- | --- | --- | --- | --- | --- | --- | --- |
| **Age** | 49 | 79 | 46 | 64 | 68 | 81 | 63 | 83 |
| **Sex** | M | F | M | M | F | M | M | F |
| **Smoking** | N | N | N | Y | N | N | N | N |
| **Drinking** | N | N | N | Y | N | N | N | N |
| **Hypertension** | N | Y | N | Y | N | Y | N | N |
| **Hyperuricemia** | N | N | N | Y | N | N | N | N |
| **Colonic polyposis** | N | N | N | Y | N | N | N | N |
| **hepatitis b** | N | N | N | Y | N | Y | N | N |
| **TNM** | Ⅳ | Ⅳ | Ⅳ | Ⅳ | Ⅳ | Ⅳ | Ⅳ | Ⅳ |
| **Tumor location** | HF | R | SF | SF | SF | C | DC | R |
| **Chemotherapy**  **regimens** | FOLFIRI | XELOX | FOLFOXIRI | FOLFIRI | FOLFIRI | FOLFOX | FOLFIRI | ERBITUX+XELOX+Tomudex |
| **Status** | Alive | Alive | Alive | Alive | Alive | Dead | Alive | Alive |

Abbreviation: SF (Sigmoid flexure), C (Cecum), DC (Descending colon), HF (Hepatic flexure), R (Rectum)
